# Wireless power transfer based on 2D routing

**DOI:** 10.1038/s41598-022-22319-5

**Published:** 2022-10-15

**Authors:** Zhouyi Wu, Haochen Yu, Dominique Schreurs, Jiangtao Huangfu

**Affiliations:** 1 Laboratory of Applied Research on Electromagnetics (ARE), College of Information Science and Electronic Engineering, Zhejiang University, Hangzhou, 310027 China; 2 ESAT-Wavecore, Department of Electrotechnical Engineering, KU Leuven, Leuven, 3001 Belgium

**Keywords:** Electrical and electronic engineering, Energy harvesting

## Abstract

In this paper, a dual-frequency wireless power transfer method is proposed, capable of achieving controllable routing and providing power through magnetic coupling resonance to various positions on a two-dimensional plane. The plane is composed of multiple power supply units with a uniform structure. Every unit has two different resonant states to switch, an activated state to power the receiver and a low-power inactive state adopted to maintain power required for state-switching. By switching and combining units in different states through wireless control circuits, directional wireless transfer of power on the plane can be realized. The circuit of power transfer through coupling is modelled and analysed. Electromagnetic simulations are conducted, followed by implementation and test of an experimental system. Both single-receiver and multiple-receiver situations are applicable in this method. The highest transmission efficiency can reach 93.3% under single receiver situation after coupling 5 units, which reveals satisfactory ability in flexibility and efficiency. Embedded in multiple application scenes, we envision further possibilities of this method such as indoor-device wireless charging and free-moving robot charging systems in factories.

## Introduction

Resonator in wireless power transfer (WPT) has been continuously studied for better transmission performance, longer transmission distance and wider transmission range^1–5^. For systems that focus on multi-dimensional motion of receiver, the use of resonators is also reported^6^. Concentric loops generate uniform magnetic field^7^ to obtain flat power transfer efficiency at any position within the transmitting area. Spherical transmitter consists of multiple transmitting coils carrying phase-shifted currents^8^ is designed to achieve uniform charging efficiency in the receiving coils. What’s more, an overlap design of four-Tx coil system is proposed^9^, where current phases of the four transmitting coils are tuned to achieve various field patterns and thus provide increased spatial freedom degrees. In contrast to works^6–8,10–12^ that only pay attention to power transmission performance and constrain movement of receivers in a scale equivalent to their own size, some research^13–15^ expands the transmitter to a range as large as the envisioned receiver free-moving space. In many cases, repeating resonant unit is adopted to form a two-dimensional transmitting plane^13^. If all the resonant units work at the same frequency, power that enters the plane will be spread around until it covers the entire plane because there is persistent power exchange between adjacent resonators. Although this ensures the availability of power supply to receivers at any position throughout the plane, it produces poor performance in terms of power transfer efficiency to the receiver. In addition, since every resonator has dissipation, when the transmission power is high, this part of loss is considerable. Therefore, controlling power flow on a plane becomes an important concern.

Typically, power flow control on a plane requires the cooperation of all units, which needs to deliver instruction to units and provide power to execute it. One strategy is to involve all the units through wired connections to establish a wired control grid beyond the wireless power transmission^16^. Another type of research focuses on the wireless realization of both power transmission and state control among the units. For every step of the receiver, the capacitance of every charging tile needs to be adjusted to tune the resonant states^17^. Therefore, a dual capacitor bank is introduced in every tile to drive the micro controller and the tuning circuit. Similarly, in order to maintain the operation of control board for real-time state adjustment, a battery is attached to each resonant unit^18^.

In these above works, while units participating in the power flow to the receiver are well utilized, the unemployed units have no access to obtain power for function implementation. Therefore wired centralized power supply or extra energy storage elements like a capacitor bank or a battery in every unit is indispensable. In this paper, unit control and power supply are achieved without wired connection or power storage in the unit. The designed unit has two resonant states at different frequencies that can be switched mutually, named as activated state and inactive state. Units in activated state form a power transfer route to transfer power to the receiver, while units out of this power flow are in inactive state that is for the power self-sustaining of state switching and communication functions. The resonant units form a two-dimensional WPT plane. By switching and combining units in different states through relay control circuits, the system is able to directionally transfer power along a route that can be flexibly adjusted according to the location of receiver. The existence of the second resonant state eliminates additional power storage components, and the redundancy of the unit circuit structure is reduced. What’s more, the number of receivers can be increased or decreased without changing circuit structure of the unit. In “The proposed WPT system”, the configuration and analysis of the proposed WPT system are first presented, followed by the mathematical model and simulation solution of the mutual inductance between coils. Then, the definition of the optimal power transfer route is given to illustrate the route selection strategy. In “Electromagnetic simulation”, electromagnetic simulations are executed. Experimental investigations are carried out to validate the proposed method in “Experimental investigations”. “Conclusion” gives a conclusion.

## The proposed WPT system

**Figure 1:**
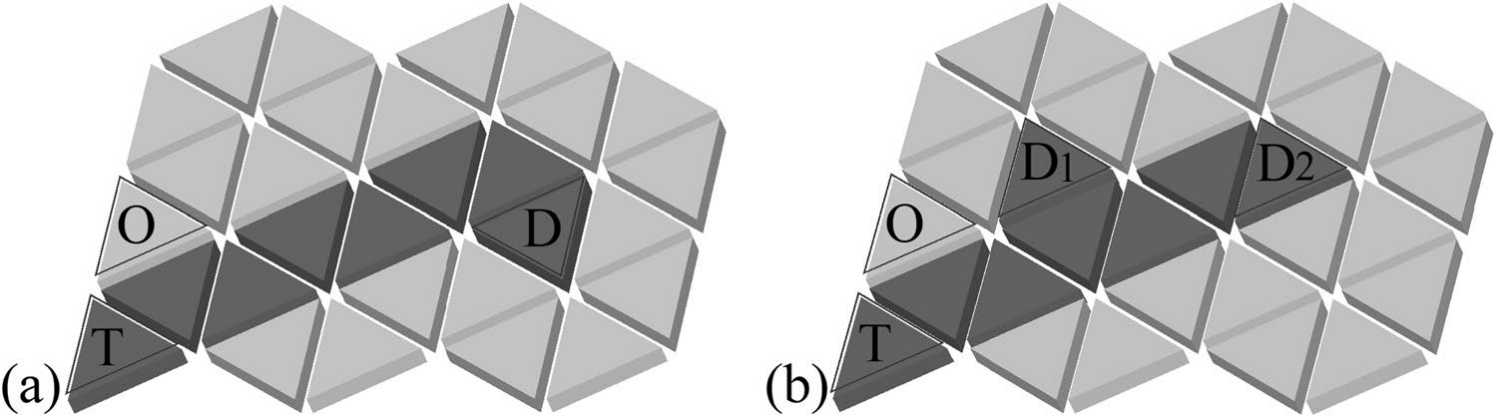
The two-dimensional power transfer plane that consists of resonant units. Dark grey triangles are activated units, and light grey triangles are inactive units. (**a**) One receiver. (**b**) Two receivers.

Figure 1a,b show the two-dimensional power transfer plane composed of state switchable resonant units. Dark grey triangles represent units in activated state, with a resonant frequency of $f_a$. Power at frequency $f_a$ empties into the plane from transmitting coil at position *T*. Light grey triangles are inactive units at resonant frequency $f_i$. Power at frequency $f_i$ comes from transmitting coil at position *O*. $D, D_1$ and $D_2$ are receivers at randomly selected locations. When there is only one receiver on the plane, as illustrated in Fig. 1a, units in activated state transfer power to the receiver through successive magnetic coupling resonance at frequency $f_a$. The whole dark grey area forms a power transfer route starting from transmitting coil *T* to the receiver *D*. When there are two receivers on the plane, Fig. 1b shows a power transfer scheme that simultaneously powers the two receivers, $D_1$ and $D_2$.

As the receiver is moving on the plane, the power transfer route needs to be dynamically adjusted. This means with every movement of the receiver, some units in inactive state will be switched to activated state, and vice versa. To realize mutual switching of the two states of a unit, the source at frequency $f_i$ is adopted to supply power to inactive units to meet the requirement of state switching, which is at a low power level. On the contrary, the source at frequency $f_a$ is usually at a high power level according to the needs of the load. The weak coupling between two flat coils results in a low power transfer efficiency, therefore capacitance is introduced to form resonance and achieve a higher efficiency. Since two circuit loops with identical resonant frequency can achieve efficient power exchange, while such exchange does not occur between two loops with differentiated resonant frequencies, power transmission among units in different states is isolated. This is also proved in the simulation and experiment parts. Additionally, even if units on the entire plane are in inactive state, the power consumption is controllable and low because the plane is distributed with power in frequency $f_i$ at a lower power level while power from source $f_a$ is blocked at the position *T* due to the difference in resonant frequency.

### System configuration with one receiver

**Figure 2:**
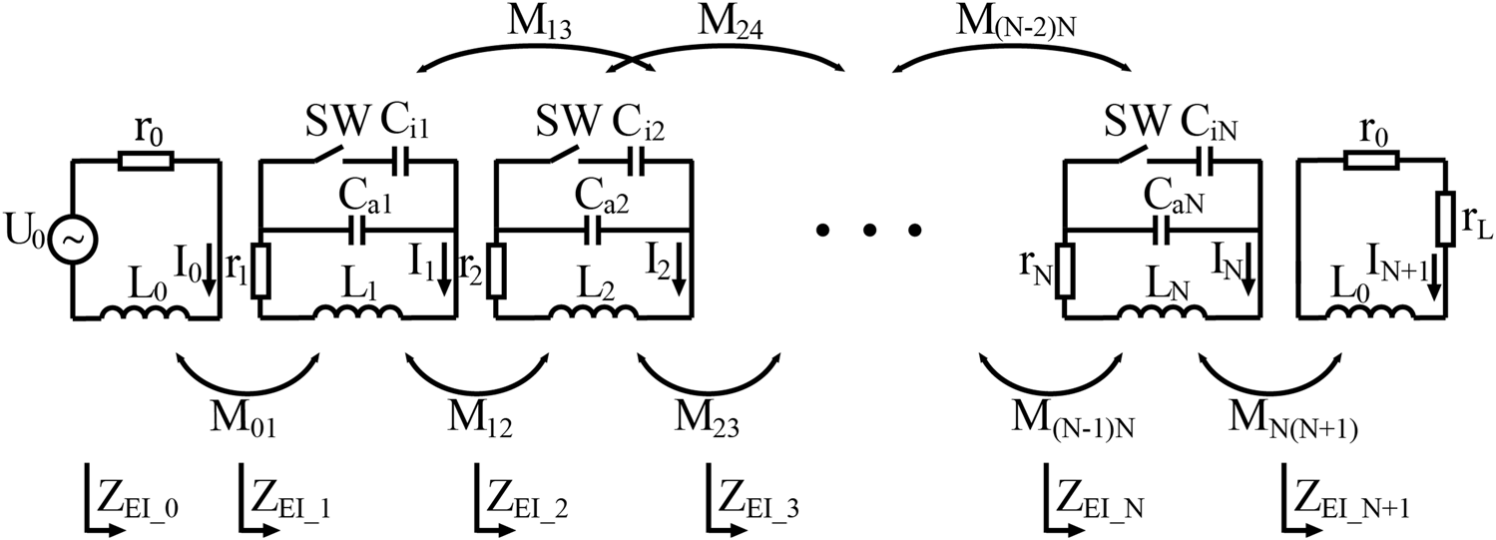
The equivalent circuit diagram of a power transfer route.

When there is a receiver on the plane, an equivalent circuit is used to analyze the power transfer route composed of *N* coupled units. Each resonant unit in Fig. 2 is modelled by an equivalent inductance $L_n$, a fixed capacitance $C_{an}$, a switchable capacitance $C_{in}$, and a resistance $r_n$ that consists of equivalent resistance of the coil and parasitic resistance of the capacitors and inductors, $n = 1, 2,\ldots , N$. In the frequency range of interest, these resistances can be regarded as constant. To a unit in activated state, the switch SW is in the open position. When the switch is closed, the unit becomes inactive. The resonant frequencies $f_a$ and $f_i$ are defined by $f_a=\frac{1}{2\pi \sqrt{L_nC_{an}}}$, and $f_i=\frac{1}{2\pi \sqrt{L_n(C_{an}+C_{in})}}$ $(n = 1, 2,\ldots , N)$, respectively.

1 $$\begin{aligned} \begin{bmatrix} U_0\\ 0\\ 0\\ \vdots \\ 0\\ 0\\ 0 \end{bmatrix} = \begin{bmatrix} r_0+j\omega L_0&{}j\omega M_{01}&{}0&{}0&{}\cdots &{}0&{}0&{}0\\ j\omega M_{01}&{}Z_1&{}j\omega M_{12}&{}j\omega M_{13}&{}\cdots &{}0&{}0&{}0\\ 0&{}j\omega M_{12}&{}Z_2&{}j\omega M_{23}&{}\cdots &{}0&{}0&{}0\\ \vdots &{}\vdots &{}\vdots &{}\ddots &{}\vdots &{}\vdots &{}\vdots &{}\vdots \\ 0&{}0&{}0&{}0&{}\cdots &{}Z_{N-1}&{}j\omega M_{(N-1)N}&{}0\\ 0&{}0&{}0&{}0&{}\cdots &{}j\omega M_{(N-1)N}&{}Z_N&{}j\omega M_{N(N+1)}\\ 0&{}0&{}0&{}0&{}\cdots &{}0&{}j\omega M_{N(N+1)}&{}r_0+r_L+j\omega L_0 \end{bmatrix} \begin{bmatrix} I_0\\ I_1\\ I_2\\ \vdots \\ I_{N-1}\\ I_N\\ I_{N+1} \end{bmatrix} \end{aligned}$$

In a power transfer route, all resonant units are in activated state. When the first unit is coupled to the transmitting coil, the equivalent circuit diagram of the power transfer route is shown in Fig. 2. Number the source circuit as 0, the load is on the $(N+1)$th loop. $L_0$ and $r_0$ represent the inductance and equivalent resistance of the transmitting and receiving coil respectively, and $r_L$ is the load resistance. $M_{pq}$ ($p = 0, 1,..., N+1, q = 0, 1,..., N+1$) represents the mutual inductance between two coils, and $M_{pq} = M_{qp}$. When analysing a resonant unit, the mutual inductance with a unit adjacent to it and one unit away from it is taken into consideration. If $\vert p-q\vert >2$, the mutual inductance between unit *p* and *q* is ignored, and $M_{pq}$ is approximated to 0.

The impedance of an activated unit is $Z_n=r_n+j\omega L_n+1/j\omega C_{an}, n=1, 2,..., N$, where $\omega =2\pi f$ is the angular frequency. The relationship of impedance, voltage and current in every loop from the transmitting coil to the receiver can be expressed as Eq. (1), where $I_n (n=0, 1,..., N+1)$ is the current. In order to improve transmission efficiency and reduce the influence of the load loop reactance on the resonant frequency of the units, a receiving coil with fewer turns is adopted. This leads to a relatively small inductive reactance, and the capacitive reactance is negligible because of the absence of stray capacitance existing among multiple turns. Therefore, the load loop is regarded as a pure resistance circuit in efficiency calculation, and the reflected impedance to previous stage is also pure resistive. When the units are at resonance, the loop impedance is $Z_n=r_n, n=1, 2,..., N$. The equivalent impedance of the *n*th $(n=1, 2,..., N-2)$ loop calculated with reflected impedance can be expressed as

2 $$\begin{aligned} Z_{EI\_n}=r_n+\frac{\omega ^2M^2_{n(n+1)}Z_{EI\_n+2}+\omega ^2M^2_{n(n+2)}Z_{EI\_n+1}}{Z_{EI\_n+1}Z_{EI\_n+2}+\omega ^2M^2_{(n+1)(n+2)}}+j\frac{2\omega ^3M_{n(n+1)}M_{n(n+2)}M_{(n+1)(n+2)}}{Z_{EI\_n+1}Z_{EI\_n+2}+\omega ^2M^2_{(n+1)(n+2)}}. \end{aligned}$$

The equivalent impedance of the rest of the loops can be expressed as

$$\begin{aligned}Z_{EI\_n}=r_n+\frac{\left[ \omega M_{n(n+1)}\right] ^2}{Z_{EI\_n+1}}, n=0, N-1, N,\\Z_{EI\_n}=r_0+r_L, n=N+1.\end{aligned}$$

Considering that only real impedance plays a role when calculating power loss, Eq. (2) can be simplified to

3 $$\begin{aligned} Z_{EI\_n}=r_n+\frac{\omega ^2M^2_{n(n+1)}Z_{EI\_n+2}+\omega ^2M^2_{n(n+2)}Z_{EI\_n+1}}{Z_{EI\_n+1}Z_{EI\_n+2}+\omega ^2M^2_{(n+1)(n+2)}}. \end{aligned}$$

To analyse the load voltage, a notation is introduced as shown in the equations below.

4 $$\begin{aligned} Z_{equi}(n,n+1)=\frac{\omega ^2M^2_{n(n+1)}Z_{EI\_n+2}}{Z_{EI\_n+1}Z_{EI\_n+2}+\omega ^2M^2_{(n+1)(n+2)}},{} \end{aligned}$$

5 $$\begin{aligned} Z_{equi}(n,n+2)=\frac{\omega ^2M^2_{n(n+2)}Z_{EI\_n+1}}{Z_{EI\_n+1}Z_{EI\_n+2}+\omega ^2M^2_{(n+1)(n+2)}},{} \end{aligned}$$

6 $$\begin{aligned} Z_{EI\_n}=r_n+Z_{equi}(n,n+1)+Z_{equi}(n,n+2),\quad n=1, 2,..., N-2. \end{aligned}$$

At the beginning and end of the power transfer route, $Z_{EI\_n}$ is denoted by

$$\begin{aligned} Z_{EI\_n}=r_n+Z_{equi}(n,n+1)=r_n+\frac{\left[ \omega M_{n(n+1)}\right] ^2}{Z_{EI\_n+1}}, \quad n=0, N-1, N. \end{aligned}$$

As the source voltage is $U_0$, the total voltage of the *n*th loop and its subsequent loops can be expressed as

7 $$\begin{aligned} U_n=U_{n-1}\cdot \frac{Z_{equi}(n-1,n)}{Z_{EI\_n-1}}, n=1, 2, N+1 {} \end{aligned}$$

8 $$\begin{aligned} U_n=U_{n-2}\cdot \frac{Z_{equi}(n-2,n)}{Z_{EI\_n-2}}+U_{n-1}\cdot \frac{Z_{equi}(n-1,n)}{Z_{EI\_n-1}},\quad n=3, 4,..., N. \end{aligned}$$

The load voltage $u_L$ can be expressed as

9 $$\begin{aligned} u_L=U_{N+1}\cdot r_L/(r_0+r_L). \end{aligned}$$

The transmission efficiency of a single-load power transfer route can be calculated as

10 $$\begin{aligned} \eta =\bigg(\frac{u_L^2}{r_L}\bigg)/\bigg(\sum \limits _{n=0}^{N}\bigg(\frac{U_n}{Z_{EI\_n}}\bigg)^2\cdot r_n+\frac{U_{N+1}^2}{Z_{EI\_N+1}}\bigg). \end{aligned}$$

In which, the numerator is the power consumption of the load. The first summation term in the denominator is the sum of the power consumed in every loop from the transmitting coil to the *N*th unit, and the second term is the power consumption of the receiving coil loop, including power consumed by the load and the equivalent resistance of the receiving coil.

### System configuration with two receivers

**Figure 3:**
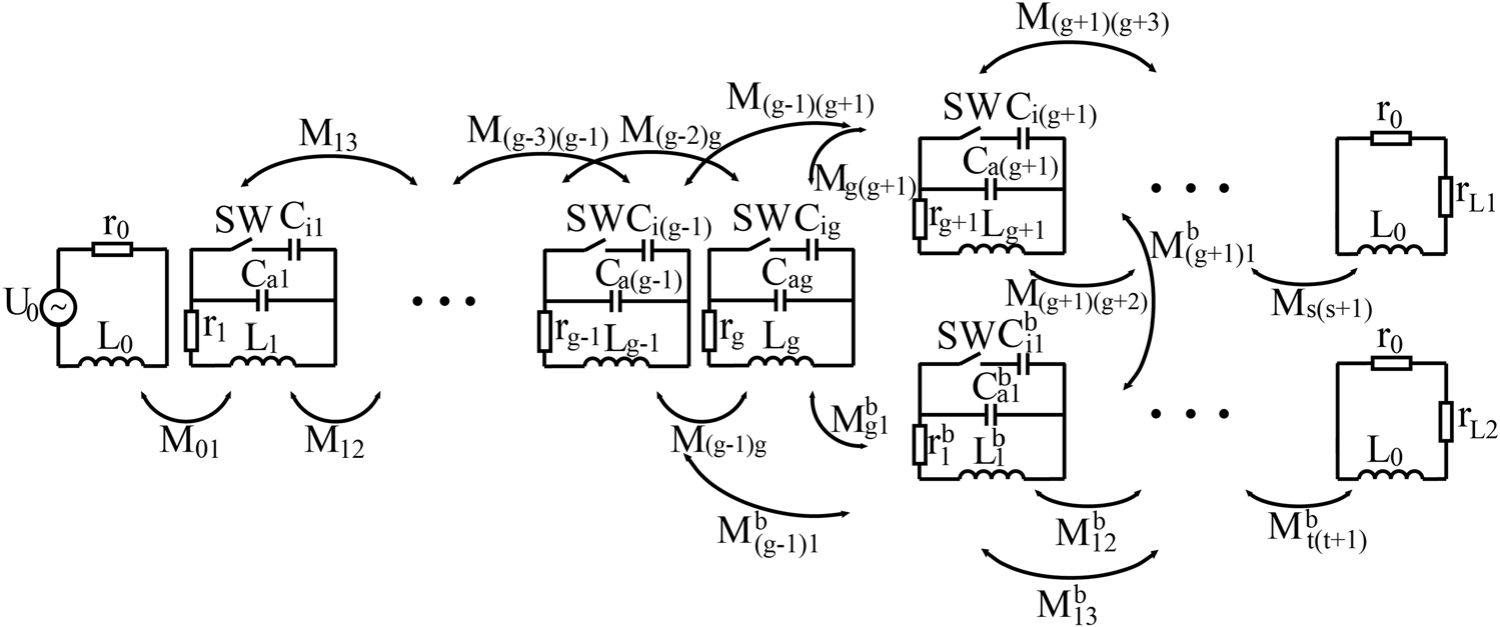
The equivalent circuit diagram of a power transfer route that splits once to reach two receivers.

When several receivers appear on the plane, the power transfer route adds branches to reach all the receivers. An example of two receivers on the plane is illustrated in Fig. 1b, where the power route splits at the fourth unit and reaches receivers $D_1$ and $D_2$ respectively. The equivalent circuit of a power transfer route that has two branches separate from the *g*th unit is shown in Fig. 3. The unit at split position exchanges power with the beginning units of the two branches simultaneously. The circuit equations from the transmitting coil to the first $(g-2)$ units are similar to Eq. (1), where accordingly $n=0, 1,..., g-2$. Moreover, the circuit equations of the units in the two branches are also treated as consistent with Eq. (1), where *n* starts from $(g+1)$ to $(s+1)$ and from 1 to $(t+1)$ respectively. To distinguish, the units in the lower branch are marked with superscript *b*. The mutual inductance associated with units in this branch is named to $M^b$, and the current of each loop is named as $I_n^b(n=1,2, ..., t+1)$. As the units numbered $(g-1)$ and *g* exchange power with units in both branches simultaneously, their equivalent impedance is obtained by building equation set with unit $(g+1)$ and $1^b$.

Up to this point, the equivalent impedance of each unit on the power transfer route has been solved according to the principle of reflected impedance. When $U_0$ is applied, the total voltage of the *n*th unit and its subsequent units in Fig. 3 conforms to Eq. (8) with $n=1, 2,..., g+1$. Then, that of the following units on the upper branch can be expressed as

$$\begin{aligned} U_n= & {} U_{n-1}\cdot \frac{Z_{equi}(n-1,n)}{Z_{EI\_n-1}}, \quad n=g+2, s+1, {} \\ U_n= & {} U_{n-2}\cdot \frac{Z_{equi}(n-2,n)}{Z_{EI\_n-2}}+U_{n-1}\cdot \frac{Z_{equi}(n-1,n)}{Z_{EI\_n-1}}, \quad n=g+3, g+4,..., s. \end{aligned}$$

The load voltage $u_{L1}$ and the transmission efficiency $\eta _1$ of the receiver on the upper branch can be expressed as

$$\begin{aligned} u_{L1}= & {} U_{s+1}\cdot r_{L1}/(r_0+r_{L1}) \\ \eta _1= & {} \bigg(\frac{u_{L1}^2}{r_{L1}}\bigg)/\bigg(\sum \limits _{n=0}^{s}\bigg(\frac{U_n}{Z_{EI\_n}}\bigg)^2r_n+\sum \limits _{n=1}^{t}\bigg(\frac{U_n^b}{Z_{EI\_n}^b}\bigg)^2r_n^b+\frac{U_{s+1}^2}{Z_{EI\_s+1}}+\frac{(U_{t+1}^b)^2}{Z_{EI\_t+1}^b}\bigg). \end{aligned}$$

Correspondingly, calculation of the $(t+1)$ loops on the lower branch conforms to the principle of upper branch. The total transmission efficiency of the power transfer route that splits once can be expressed as

11 $$\begin{aligned} \eta =\eta _1+\eta _2=\bigg(\frac{u_{L1}^2}{r_{L1}}+\frac{u_{L2}^2}{r_{L2}}\bigg)/\bigg(\sum \limits _{n=0}^{s}\bigg(\frac{U_n}{Z_{EI\_n}}\bigg)^2r_n+\sum \limits _{n=1}^{t}\bigg(\frac{U_n^b}{Z_{EI\_n}^b}\bigg)^2r_n^b+\frac{U_{s+1}^2}{Z_{EI\_s+1}}+\frac{(U_{t+1}^b)^2}{Z_{EI\_t+1}^b}\bigg). \end{aligned}$$

In which, the numerator is the power consumption of two loads, and the denominator is the sum of power consumption of all the loops involved in power transfer in the dual-receiver situation. For the relay plane composed of triangular units, each additional branch reaching a receiver requires a route split at a unit. The equivalent circuit of a power transfer route with more receivers on the plane can be derived according to the principle of the route that splits once.

### Mutual inductance

**Figure 4:**
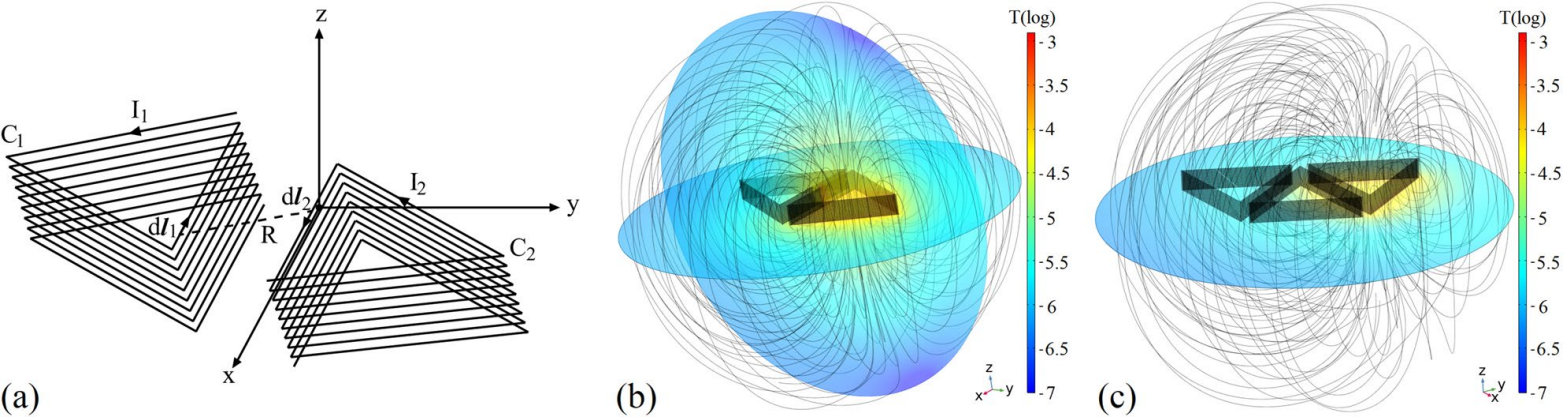
(**a**) The mutual inductance model of two helical triangular coils. (**b**) Simulation results of coupling between two adjacent coils. (**c**) Simulation results of coupling of three coils.

Stereo helical coil is adopted as part of the resonant unit, because it provides better coupling effect compared to a flat looped coil. Considering that a triangle has three sides, one of which is used to couple with the unit in the previous stage to acquire power, when it comes to power transfer routing, the remaining two sides of the triangular unit lead to a binary choice. This produces the lowest route selection complexity of the system. Additionally, a triangle has the longest side coupling length compared with other polygons with the same perimeter. Therefore, helical triangular coils are used in Fig. 4a. Selection of the optimal power transfer routes will be discussed in the next part.

As illustrated in Fig. 4a, take line elements $d\varvec{l}_1=dx{\widehat{i}}+dy{\widehat{j}}$ and $d\varvec{l}_2=dx'{\widehat{i}}+dy'{\widehat{j}}$ on two coils $C_1$ and $C_2$, respectively, the distance between $d{\varvec{l}}_1$ and $d{\varvec{l}}_2$ is denoted by *R*. According to Neumann’s formula, the mutual inductance between two units with the number of turns $K_1=K_2=K$ can be approximated by

12 $$\begin{aligned} M=\frac{K^2\mu _0}{4\pi }\oint _{C_2}\oint _{C_1}\frac{d\varvec{l}_1\cdot d\varvec{l}_2}{R}=\frac{K^2\mu _0}{4\pi }\bigg(\oint _{C_2}\oint _{C_1}\frac{dxdx'}{\sqrt{(x-x')^2+(y-y')^2}}+\oint _{C_2}\oint _{C_1}\frac{dydy'}{\sqrt{(x-x')^2+(y-y')^2}}\bigg). \end{aligned}$$

The value of mutual inductance between two coils is simulated in COMSOL Multiphysics, with $K=13$. The cross section of the metal strips is 2 mm$\times$2 mm, and each layer is the same equilateral triangle with a side length of 270 mm. According to the optimization results of the multi-unit electromagnetic simulation, the spacing between each layer is set to 2 mm, and the gap width between two coils is 22 mm in Fig. 4b. The mutual inductance is 4487.9 nH. In the previous part, the mutual inductance of an activated unit with units adjacent to it and one unit away from it are considered. As illustrated in Fig. 4c, the mutual inductance of two coils when another coil exists in the middle is simulated as well. This mutual inductance is 923.64 nH, which is $20.58\%$ of the mutual inductance between adjacent coils. Therefore, on a power transfer route, the mutual inductance of a unit with another unit one unit away from it is not negligible. The mutual inductance that exists between a transmitting or receiving coil and a unit coil in Fig. 2 is 2677.3 nH.

### Optimal routes

**Figure 5:**
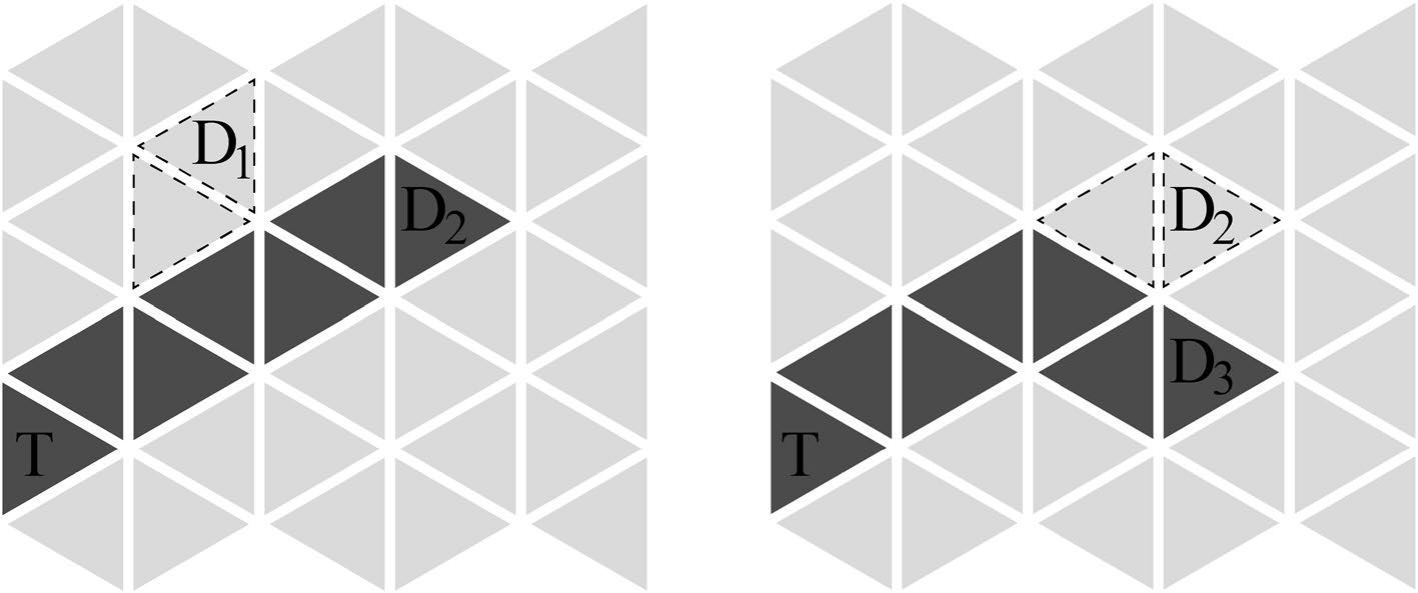
Optimal routes when the receiver moves from positions $D_1$ to $D_2$ and $D_2$ to $D_3$.

Once the receiver is on the plane, the route that passes the least amount of units from transmitting coil to the receiver is the shortest and is defined as the optimum. Since a triangular coil is adopted for a unit, every stage of the power transfer route requires a binary choice. When there are multiple shortest routes, ignore the influence of the surrounding inactive units, these routes are equivalent in terms of transmission efficiency according to the equivalent circuit. In this case, the optimal route is selected randomly from them. When the receiver moves around, as illustrated in Fig. 5, the optimal power transfer route will be recalculated in order to minimize transmission cost at frequency $f_a$, with the change of unit states in related locations. In this process, the optimal route from the source to the next position is defined as the shortest route with the least number of units that need to be switched. Figure 5 shows the selected optimal routes when the receiver moves from location $D_1$ to $D_2$ and $D_2$ to $D_3$. The triangles with a dashed line side are units turned from activated into inactive due to route switching. The dark grey area from *T* to $D_2$ is the only shortest route. When the location is $D_3$, there are three shortest routes from *T* to $D_3$, where the dark grey area in Fig. 5 is the one with the least number of units to switch state.

Bypassing some specific positions is also under consideration if there are some places on the plane that should be avoided. When several receivers appear on the plane, the criterion of the least unit amount remains available for optimal routing. By and large, comparing the total power consumption of the routing schemes that transfer power from transmitting coil to receivers, the candidate with the lowest cost is taken.

## Electromagnetic simulation

**Figure 6:**
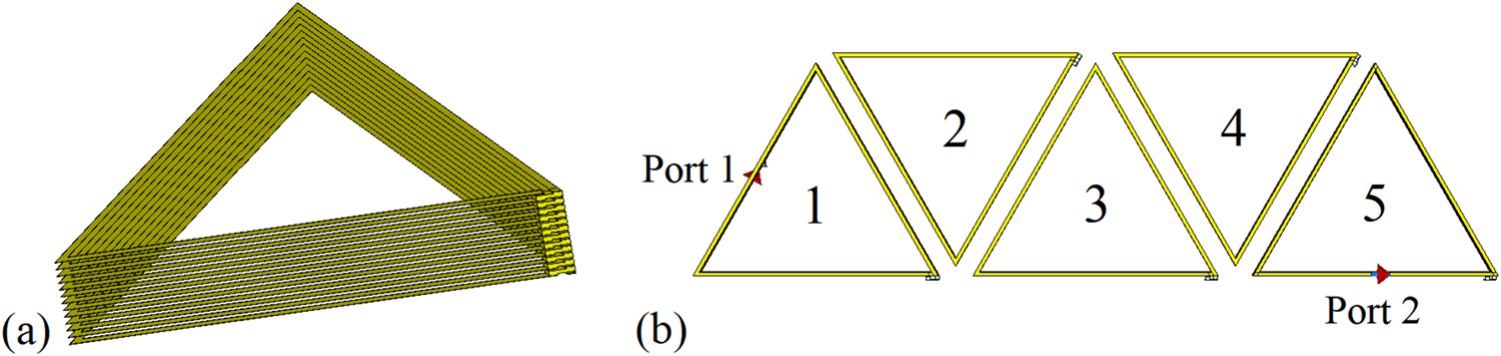
(**a**) A thirteen-layer helical triangular coil. (**b**) Simulation model of five units arranged in line, with a transmitting coil, a receiving coil and two ports.

**Figure 7:**
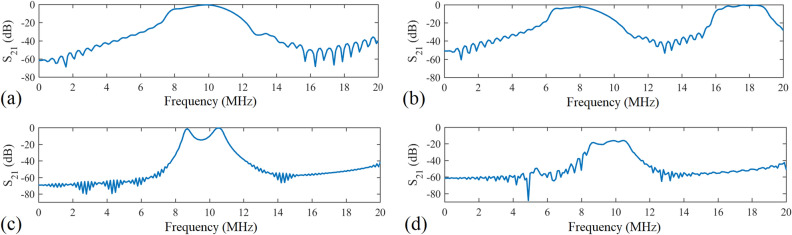
Electromagnetic simulation results. (**a**) $S_{21}$ of five activated units. (**b**) $S_{21}$ of five inactive units. (**c**) $S_{21}$ of activated units 1, 2, 4, 5 and an inactive unit 3. (**d**) $S_{21}$ of activated units 1, 4, 5 and inactive units 2, 3.

The simulation model of the helical triangular coil is shown in Fig. 6a. For an inactive unit, a lumped capacitance $C_{in}=10 pF$ is introduced to shift the resonant frequency. When it comes to a unit in activated state, the capacitive part of the resonance circuit is the parasitic capacitance $C_{an}$ of the coil. The parameters in Fig. 2 are, inductance $L_n=16\mu H$, capacitance $C_{an}=19pF$, and resistance $r_n=0.25\Omega$. The transmitting and receiving coils are modelled as two equilateral triangles with the same side length as the helical coil, with $L_0=3.4\mu H$, $r_0=0.02\Omega$. Figure 6b suggests that each coil is coupled to the previous and the next units, with a transmitting coil connected to AC source and a receiving coil connected to the load. The simulation port acts as the structure prepared for the connection to the source and load.

Initially, the system is modelled as five units arranged in line and numbered from 1 to 5, as shown in Fig. 6b. The transmitting and receiving coils are vertically 1.5 mm away from the units. The first simulation uses five activated units to form a power transfer route in CST Microwave Studio. The resonant frequency of activated units $f_a$ is obtained around 9.9 MHz in Fig. 7a. Then, the units are turned into inactive ones. The resonant frequency of inactive units $f_i$ is 8 MHz in Fig. 7b, shifted by 1.9 MHz compared to activated state. As shown in Fig. 7a, the optimum result of $S_{21}$ after coupling through 5 activated units at 9.9 MHz is -0.5 dB, indicating a high AC transmission efficiency of 89.1%. By contrast, in Fig. 7b when power is coupled through 5 inactive units at 8 MHz, the whole system still exhibits an acceptable efficiency of 57.5%, -2.4 dB. Therefore, even if the units are in inactive state, power at frequency $f_i$ can be well utilized. Under this setup the insertion loss at 9.9 MHz is 97.2%, with the $S_{21}$ falling to -15.6 dB. Next, state of units numbered 1, 2, 4, 5 in Fig. 6b is switched to activated, with the inactive unit number 3 in the middle. The power transmission efficiency to the receiving coil drops to $-12$ dB (6.3%) at 9.9 MHz, as shown in Fig. 7c. Continue to switch unit number 2 to inactive state, according to the result in Fig. 7d, the $S_{21}$ is $-16.3$ dB at 9.9 MHz, equivalent to a transmission efficiency of 2.3%. The transmission of power at resonant frequency $f_a$ is dramatically blocked. So there is sufficient isolation between two units in different states, which verified the high concentration of power transfer route in this method. The simulation results of 5 units in Fig. 6b with different states is summarized in Table 1.

**Table 1:** Simulation results of transmission efficiency of 5 units in various states.

Source frequency (MHz)	Activated units	Inactive units	Transmission efficiency ($\%$)
8	/	1, 2, 3, 4, 5	57.5
9.9	1, 2, 3, 4, 5	/	89.1
9.9	1, 2, 4, 5	3	6.3
9.9	1, 4, 5	2, 3	2.3

**Figure 8:**
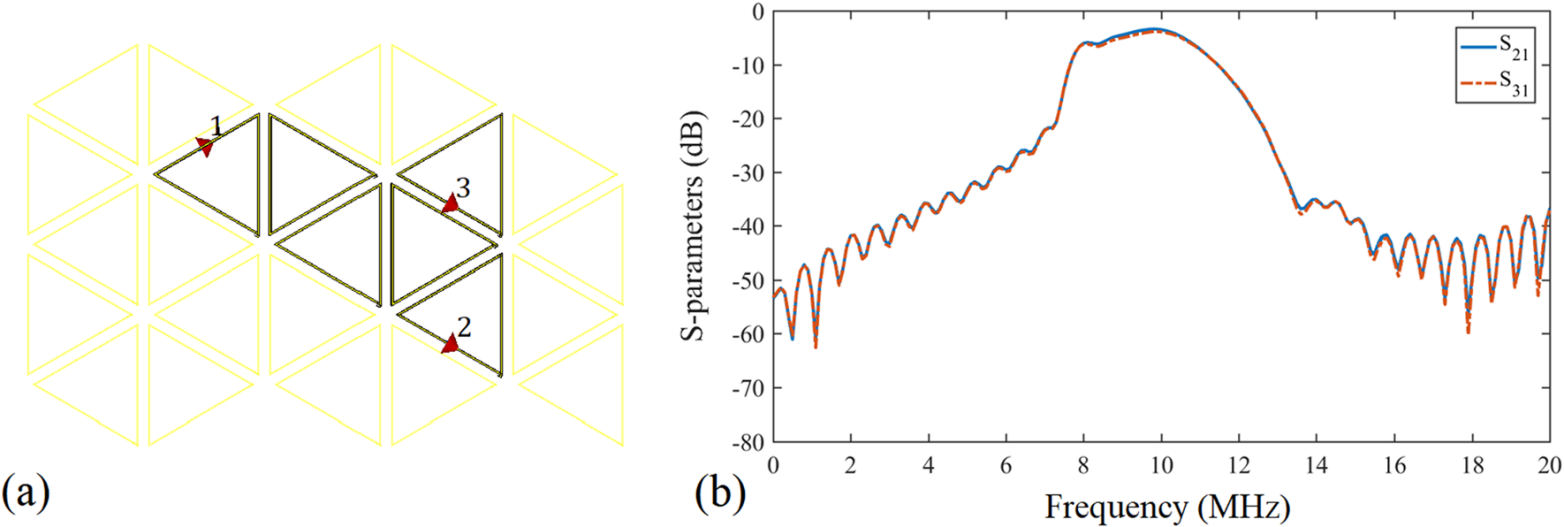
(**a**) A two-receiver power transfer plane model. The highlighted triangles are activated units, and inactive units are in light yellow. (**b**) $S_{21}$ and $S_{31}$ of the power transfer route in electromagnetic simulation.

Finally, the whole two-dimensional WPT plane is simulated under the circumstance of multiple receivers. In Fig. 8a, $port\,1$ is set as the power source at frequency $f_a$, and $port\,2$ and 3 are loads. The highlighted power transfer route consists of 6 activated units, surrounded by inactive units in light yellow. Figure 8b shows the simulation results of transmission from $port\,1$ to $port\,2$ and $port\,3$. At 9.9 MHz, the magnitude of $S_{21}$ is $-3.4$ dB, and $S_{31}$ is $-3.9$ dB. Compared to the one-receiver curve in Fig. 7a, where the power transfer efficiency at 9.9 MHz is 89.1%, in two-receiver situation the efficiency at 9.9 MHz is 45.7% in $port\,2$ and 40.7% in $port\,3$, revealing an overall performance of 86.4%. According to the simulation parameters, the numerical calculation result of the power transfer efficiency in the case of a single receiver after coupling of 5 units is $96.96\%$. The internal resistance of power source is 10 $\Omega$. In the case of two receivers, when there are 6 activated units in resonance, the theoretical efficiency is $\eta _1=\eta _2=48.94\%$, with a total efficiency of $97.89\%$.

## Experimental investigations

**Figure 9:**
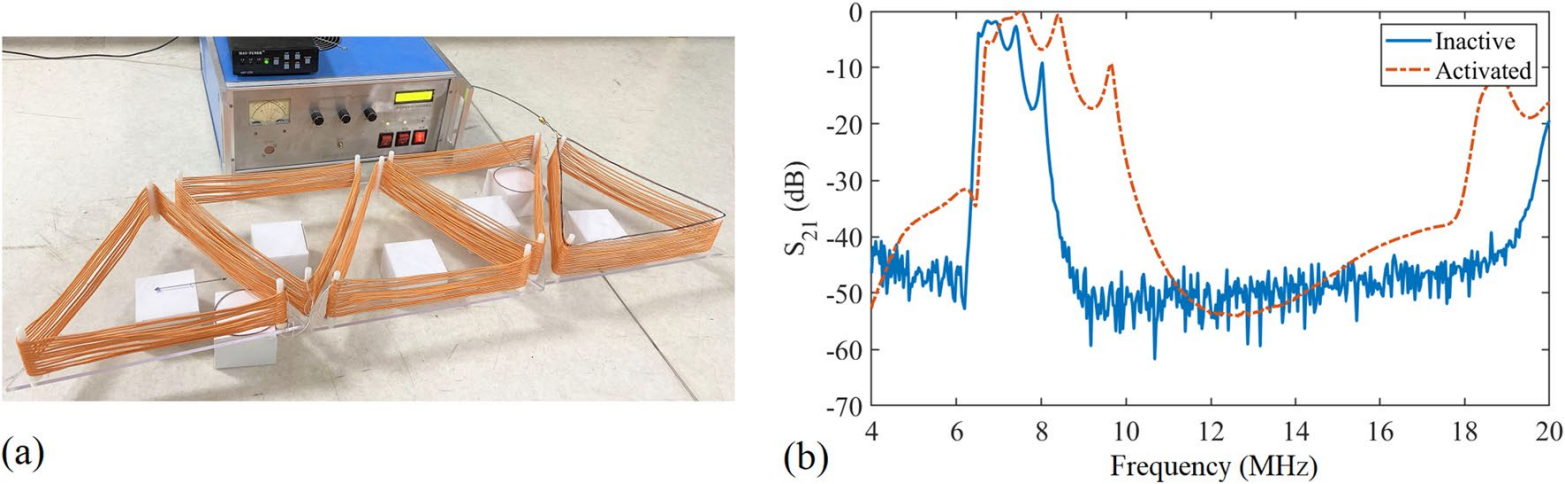
(**a**) Five resonant units with a power source and a transmitting coil. (**b**) Experimental results of $S_{21}$ of five inactive units and five activated units.

The construction of experimental system follows the physical parameters in simulation section. The working frequencies lead to a wavelength much larger than the unit size, so as to reduce radiation loss and realize magnetic coupling among coils to transfer energy. Values of the two resonant frequencies, $f_a$ and $f_i$, are first tested through five lined up units in Fig. 9a. The metal strips of the helical triangular coil in the simulation model are replaced by silk-covered copper litz wire with a diameter of 2 mm as the inductance in the experimental system. This results in a difference in impedance between coils in simulation model and in experiment. The litz wire is composed of 200 individually insulated fine strands, which help to reduce the skin effect and proximity effect losses. The frame that gives structural support is composed of an equilateral triangle acrylic sheet and three M8 plastic screws. Distance between two screws is 270 mm, and each screw is 11 mm away from the closest edge of the triangle sheet. Starting from the bottom of a screw to the top, silk-covered litz wire wraps around the outside of three screws 13 times with a uniform spacing of 2 mm between each turn. The two ends of the coil are connected to a 10 pF ceramic capacitor and the normally closed side of a relay module in series. The transmitting and receiving coils are placed on the left and right ends of five units, and they are made by enamelled copper wire with a diameter of 1.1 mm. Without external signal, the switches are closed and units are inactive. The measured $S_{21}$ is drawn in the solid line in Fig. 9b. The optimum appears at the resonant frequency of 6.7 MHz with -1.7 dB, equal to an efficiency of 67.6%. This is the frequency of $f_i$, with a deviation of 1.3 MHz compared to the electromagnetic simulation result due to the influence of various parameter changes in system elements. Next, $S_{21}$ of five activated units is measured and the result is shown as the dashed line in Fig. 9b. The activated state has an optimal transmission efficiency of -0.6 dB at 8.4 MHz, 1.5 MHz lower than the result in Fig. 7a. This frequency is set as $f_a$.

**Figure 10:**
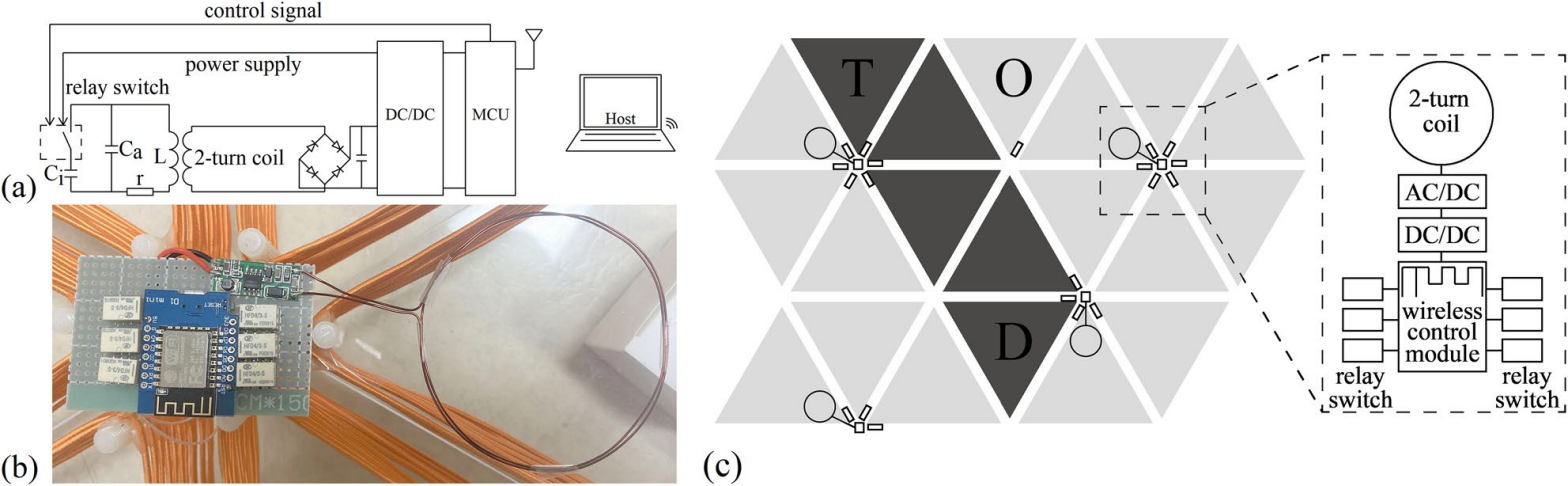
(**a**) The structure of system control. (**b**) Relay control circuit. (**c**) A schematic of the control system. The dashed box on the right is an enlarged view of one control circuit and six relay switches.

A wireless system control structure is introduced to manage the state of units, as drawn in Fig. 10a. A computer is configured as a TCP wireless server. MCUs are wirelessly connected to the computer through Wi-Fi and act as TCP clients whose pin outputs are controlled through local IP addresses. The relay switches are normally closed if there is no control signal input. When the plane is in operation, the unit under the transmitting coil *O* keeps inactive, and the unit under the transmitting coil *T* is always in activated state. As illustrated in Fig. 10b,c, ESP8266s are adopted as MCUs for relay control and a 2-turn coil is introduced to couple with triangular coils. Every 2-turn coil of the relay control circuit is placed under a unit. When the above unit is activated, the relay control circuit is powered by activated state. And when the unit is switched to inactive, the relay control circuit is powered by inactive state. The coupled AC power is supplied to the relay switch and ESP8266 after AC/DC and DC/DC conversion, so that the relay control circuit is self-powered when the plane is in operation. The 2-turn coil is made by 1.1 mm enamelled copper wire with a diameter of 72 mm. Since there can be six equilateral triangles around one vertex, when units are pieced together, each ESP8266 provides signal for several relay switches around a common vertex.

**Figure 11:**
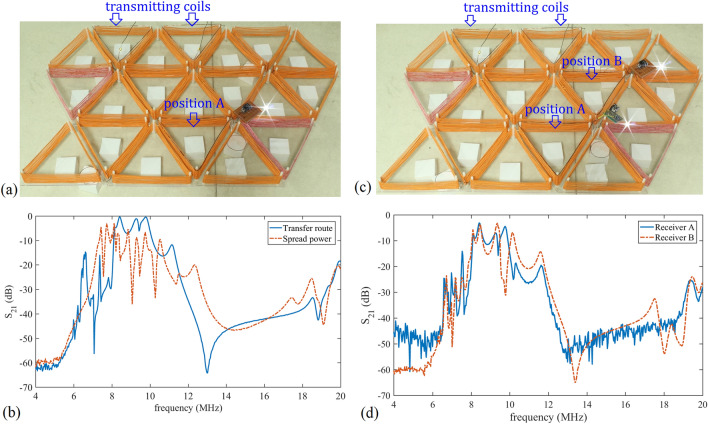
(**a**) The 20-unit system. The receiving coil is in position *A*. (**b**) $S_{21}$ of the power transfer route solution (solid line) and the spreading power solution (dashed line) in one-receiver experiment. (**c**) The 20-unit system with two receiving coils in position *A* and *B*. (**d**) The experimental results of $S_{21}$ and $S_{31}$ in two-receiver experiment.

Further, a 20-unit system is achieved in Fig. 11a. Two power sources feed AC power into the plane via two transmitting coils, with frequency $f_a$ on the left and $f_i$ on the right, and the load harvests power from a receiving coil. Since coil coupling is a linear process, the input single-frequency power will not be shifted to other frequencies during the transmission along the power transfer route. Initially, all the units are in inactive state. When the receiving coil is in position *A* in Fig. 11a, units on the optimal power transfer route are switched from inactive state to activated state. The measured $S_{21}$ is shown in the solid line in Fig. 11b. The optimum appears at frequency $f_a$ of 8.4 MHz with -0.3 dB, equivalent to a transmission efficiency of 93.3%. For comparison, all units are then switched to activated state, allowing power to spread around on the plane instead of transferring along a power transfer route. The transmission measured from the left transmitting coil to position *A* is drawn with dashed line in Fig. 11b. $S_{21}$ at the resonant frequency 8.4 MHz is −15.3 dB. It can be seen that only 3% of the power is transmitted to the receiver through this power spread method.

The power source used in the experiment has an output range of 0–100 W with an RF tuner in a frequency range of 2–30 MHz. For inactive units, the output frequency is set to 6.7 MHz, and for activated units it is 8.4 MHz. Before being sent to transmitting coils, the output from the source is filtered by RF tuner to remove high-order harmonics. In this 20-unit system, a total of four ESP8266 MCUs are adopted. The power consumption of every ESP8266 is 180 mW. A relay switch requires constant energy to maintain its operation, and external signals to change its open and close state. For every relay switch, 10 mW is required to keep it in working mode, and this power is increased to 120 mW when it needs to switch states. The coupling efficiency between the 2-turn coil and an inactive unit at $f_i$ is $-4.2$ dB, so when the units above the 2-turn coils are all inactive ones, the power source $f_i$ needs to provide at least 6 W to achieve switch-state controlling of the 20 units on the whole plane. Oppositely, when 2-turn coils are coupling with activated units in a power transfer route, 7 W of extra power is required in addition to the amount provided to the receiver. The coupling efficiency between the 2-turn coil and an activated unit at 8.4 MHz is $-5.5$ dB. Moreover, the existence of the 2-turn coil has little disturbance to the resonant frequency of the experimental system. If the receiving coil is connected to a 1 W LED after AC/DC conversion, as shown in Fig. 10a, the LED is lightened up when the $f_a$ source output power is higher than 156 mW. Gradually increase the $f_a$ power to more than 10 W, the AC power efficiency keeps in 93.3%.

Under this setup the second receiver is added to position *B* in Fig. 11c, so that the optimal route is consistent with that of the simulation. The measured transmission curves of the two receivers are drawn in Fig. 11d. The optimum is at frequency $f_a$ of 8.4 MHz, where $S_{21}$ is $-3.1$ dB and $S_{31}$ is $-3.6$ dB. When the $f_a$ output power is higher than 325 mW, the two receivers work. The power efficiencies of receiver *A* and *B* are 49% and 43.7%, respectively, which is consistent with the electromagnetic simulation results. The simulation and experimental results in terms of frequency and transmission efficiency, as well as calculation of theoretical model are summarized in Table 2. The theoretical model is confirmed by the simulation result and experimental measurement.

**Table 2:** Performance summary.

Part	Frequency		Efficiency			
	$f_a$ (MHz)	$f_i$ (MHz)	1 receiver/5 units (%)	2 receivers/6 units		
				$r_{L1}$ (%)	$r_{L2}$ (%)	*Total* (%)
Theoretical model	–	–	96.96	48.94	48.94	97.89
Simulation	9.9	8	89.1	45.7	40.7	86.4
Experiment	8.4	6.7	93.3	49	43.7	92.7

## Conclusion

This paper has proposed a two-dimensional routing WPT system based on dual-frequency coupling resonance to obtain directed and concentrated power transmission. By splitting the plane into triangular units, this system enables flexible routing and powering a receiver at arbitrary location. What’s more, the introduced wireless relay control circuit simultaneously realizes the dynamic switching of the power transfer route and the self-powering of the circuit itself. The equivalent circuit of the power transfer route and the mathematical model of mutual inductance are built and analysed. Electromagnetic simulation and experiment are performed to verify the method. In this 20-unit experimental system, an AC power transfer efficiency of 93.3% can be achieved between transmitting and receiving coils by coupling resonance through 5 units. The results demonstrate that dual-frequency resonant units can be utilized to transfer and isolate power on a two-dimensional plane according to requirements. In the future, intelligent routing that helps to improve system efficiency and stability will be studied.

## Data Availability

The data produced and analyzed during the current study are available from the corresponding author on reasonable request.
